# Association of mainly vegetarian and vegan diets with loneliness, social isolation and social withdrawal in a German population survey

**DOI:** 10.1371/journal.pone.0353869

**Published:** 2026-07-15

**Authors:** André Hajek, Razak M. Gyasi, Supa Pengpid, Karl Peltzer, Karel Kostev, Pinar Soysal, Ashwin Kotwal, Lee Smith, Dong Keon Yon, Hans-Helmut König

**Affiliations:** 1 Department of Health Economics and Health Services Research, University Medical Center Hamburg-Eppendorf, Hamburg Center for Health Economics, Hamburg, Germany; 2 African Population and Health Research Center, Nairobi, Kenya; 3 National Centre for Naturopathic Medicine, Faculty of Health, Southern Cross University, Lismore, New South Wales, Australia; 4 Department of Health Education and Behavioral Sciences, Faculty of Public Health, Mahidol University, Bangkok, Thailand; 5 Department of Public Health, Sefako Makgatho Health Sciences University, Pretoria, South Africa; 6 Department of Healthcare Administration, College of Medical and Health Science, Asia University, Taichung, Taiwan; 7 Department of Psychology, University of the Free State, Bloemfontein, South Africa; 8 Department of Medical Research, China Medical University Hospital, China Medical University, Taichung, Taiwan; 9 University Hospital Marburg, Philipps-University Marburg, Marburg, Germany; 10 Department of Geriatric Medicine, Faculty of Medicine, Bezmialem Vakif University, Istanbul, Turkiye; 11 Division of Geriatrics, Department of Medicine, University of California, San Francisco, California, United States of America; 12 Geriatrics, Palliative, and Extended Care Service Line, San Francisco Veterans Affairs Medical Center, San Francisco, California, United States of America; 13 Centre for Health, Performance and Wellbeing, Anglia Ruskin University, Cambridge, United Kingdom; 14 Department of Public Health, Faculty of Medicine, Biruni University, Istanbul, Turkey; 15 Center for Digital Health, Medical Science Research Institute, Kyung Hee University College of Medicine, Seoul, South Korea; Ghent University: Universiteit Gent, BELGIUM

## Abstract

**Aim:**

There is a very limited number of studies examining how diet is associated with social factors. The aim of this study was therefore to examine the association between type of diet and loneliness, social isolation and social withdrawal.

**Methods:**

Cross-sectional data from the general adult population in Germany were used. The sample included 5,000 individuals aged 18–74 years. To quantify loneliness, the De Jong Gierveld tool was used. Social isolation was measured using the Lubben Social Network Scale and Bude/Lantermann tool. Social withdrawal was operationalized using the 25-item Hikikomori Questionnaire. Three types of diet were considered (mainly vegetarian; mainly vegan; neither vegetarian nor vegan, reflecting an omnivorous diet). The analysis was adjusted for sociodemographic, lifestyle-related, health-related and mental health-related covariates in multiple linear regressions.

**Results:**

Overall, 15.4% of the individuals reported following a mainly vegetarian diet and 3.5% of the individuals surveyed followed a mainly vegan diet, with the remaining participants (81.1%) following an omnivorous diet. When adjusting for sociodemographic, lifestyle-related and health-related factors, individuals following a mainly vegetarian diet had significantly higher levels of perceived social isolation and social withdrawal compared to individuals following an omnivorous diet. However, these significant associations disappeared when adjusting for mental health-related covariates. Mainly vegan eaters feel more isolated and withdraw more from society compared to individuals following an omnivorous diet, even in the fully adjusted model.

**Conclusion:**

Following a mainly vegan diet was associated with perceived social isolation and social withdrawal, even when adjusting for sociodemographic, lifestyle-related, health-related and mental health-related covariates. However, the variance explained by diet type was minimal (R^2^ close to zero and partial eta^2^ < 0.01), indicating limited practical relevance.

## 1 Introduction

Different types of diet exist. While many humans consume fish and meat (omnivores), there is an increasing number of vegetarians (i.e., individuals who abstain from fish and meat) and vegans (individuals abstaining from products that are wholly or partially derived from animals, such as dairy products or eggs) in Germany. According to representative figures, 9% of individuals define themselves as vegetarian, with 3% of the general adult population in Germany defining themselves as vegan in 2023 [1].

In Germany, people follow a vegetarian or vegan diet for various reasons, such as health reasons, social/moral motivations, religion, and/or sustainability reasons [2]. Vegetarian products are widely available in Germany. The availability of vegan alternatives has also increased in Germany over several years [3]. However, there is tentative evidence suggesting that vegetarian dishes are more common than vegan dishes in German restaurants [4].

In terms of the relationship between vegetarian/vegan diets and health, previous research has shown that following a vegetarian diet is associated with favorable health outcomes such as better cognitive functioning, a lower risk of being obese, better cardiovascular health and higher life expectancy [5–8]. However, a divergent picture emerges when considering other health outcomes, particularly those related to mental health. A meta-analysis showed that meat consumers had lower depression and anxiety compared to vegans [9]. However, in contrast to this, a systematic review [10] concluded that the “evidence on the effect of vegetarian and vegan diets on depression is contradictory” (p. 27).

While there has been a number of studies related to health factors and diet, there is a very limited number of quantitative studies examining how the type of diet is associated with social factors such as loneliness (negative state referring to the perceived discrepancy between actual and desired social relationships [11]), objective social isolation (lack of social activities, see [12]), perceived isolation (feeling of not belonging to the society) [13] and social withdrawal (a tendency to avoid social activities and to isolate from important peer groups [14]). More precisely, the extant studies have relied on qualitative research methods. For example, a qualitative study from Chile showed that some vegans experienced loneliness because “the rest of the society does not share the same questioning, and therefore, they feel like outsiders” (p. 571) [15]. Another qualitative study from Norway also reported experiences of isolation and frustration among vegans [16]. A further qualitative study among older vegan women in the USA showed that some reported stressed social interactions and a lack of support provided by doctors [17]. Another qualitative study used an autoethnographic approach [18] and described that a vegetarian lifestyle can be associated with feelings of loneliness. While these qualitative studies are valuable for exploring the relationship between dietary patterns and feelings of loneliness in detail, there is a great lack of quantitative studies exploring these topics.

Such quantitative studies are required to provide findings that are generalizable to entire populations. Moreover, such quantitative knowledge is important in identifying individuals (depending on their type of diet) who are susceptible to loneliness, isolation, and social withdrawal (which are in turn associated with negative health outcomes such as morbidity and mortality [19,20]). Subsequently, this could assist in the prevention of marginalization of specific social groups adhering to particular dietary regimens. It is important to acknowledge the significance of marginalization, as it has the potential to diminish trust and respectful interaction among diverse groups. This has the potential to compromise social cohesion and give rise to societal polarization [21,22].

In accordance with previous qualitative research mentioned above [15,16,18], we generally assume that the type of diet one has may be a factor that can lead to loneliness, isolation and marginalization. For example, if there are no vegetarian/vegan alternatives at a barbecue where meat and fish are served, individuals following a vegetarian or vegan diet could feel marginalized [23]. Even if such vegetarian/vegan alternatives are offered, such individuals may feel lonely or marginalized if they are one of the few people eating vegetarian/vegan alternatives and are exposed to incomprehension, disrespectful comments or ridicule from others – as described by a previous qualitative cross-sectional survey of 1,053 Australian men living in Sydney, Australia [24]. This could also lead to social withdrawal and isolation [24]. However, such potential feelings are most likely not limited to barbecues. Other social situations, such as the choice of restaurant when going out to eat with friends or relatives or a possible lunch with work colleagues in a canteen may also be important for a potential association between type of diet and social outcomes (see also: [23]). Vegetarians/vegans may feel a burden to colleagues and family members [18] since others may need to choose restaurants in line with the eating habits of the vegetarians/vegans. As a result, vegetarians/vegans may end up eating alone [18]. This may contribute to feelings of loneliness, isolation, and social withdrawal.

According to both stigma theory and minority stress theory [25,26], vegetarians and vegans may experience stigmatization due to social prejudice and a lack of acceptance [27,28]. For example, a previous qualitative study suggested that vegan stigma is an obstacle that prevents people from switching to a plant-based diet [28], which ultimately may lead to feelings of exclusion. Negative social evaluation of oneself and attempts to protect oneself from discrimination can reinforce feelings of isolation and withdrawal [29–31]. Furthermore, the stress caused by dealing with prejudice can hinder social participation and exacerbate feelings of loneliness and isolation [29].

In addition, vegans/vegetarians may feel anger toward others because, at least from their perspective, those who eat meat ignore or accept animal suffering [15]. In the worst-case scenario, this could lead to friendships being ended or individuals distancing themselves from each other (or, being shunned by others, for example, due to the fear of starting arguments with other guests about animal husbandry or animal suffering). This could lead to feelings of loneliness, isolation and social withdrawal. Additionally, feelings of embitterment may be present among vegans/vegetarians [32], which are in turn associated with loneliness [33].

It should be mentioned that special vegetarian and vegan restaurants exist in Germany, particularly in larger cities. Such restaurants could strengthen feelings of togetherness among those who do not eat meat [18]. Vegetarians and vegans could see themselves as part of a growing community where they can exchange ideas with like-minded people (see: [34]). These could in turn counter feelings of loneliness, isolation and withdrawal. However, overall, we assume that following vegetarian and especially vegan diets is associated with unfavorable social outcomes (in terms of higher levels of loneliness, social isolation and social withdrawal). This association could be explained by potential experiences of marginalization at meal opportunities and potential negative feelings, such as anger.

In sum, due to the limited knowledge available that is based on quantitative studies, the aim of this study was to examine the association of type of diet with loneliness, social isolation and social withdrawal, based on quantitative data from the general adult population in Germany.

## 2 Materials and methods

### 2.1 Sample

Data were gathered from a sample of 5,000 individuals living in Germany. The individuals were between 18 and 74 years old. The data collection was conducted via an online survey by the market research company Bilendi (https://www.bilendi.co.uk/) and occurred in late summer 2023 (i.e., August and September). To ensure the sample was representative of the general adult population in Germany, a quota-based online sampling method was used, considering age, gender and federal state. This study followed the STROBE guidelines. The market research company Bilendi carefully checks its online panel on an ongoing basis, including rigorously removing duplicates and respondents with inconsistent answers. In this study, Bilendi also performed variance checks to detect random response patterns and exclude inconsistent answers. They compared survey responses with previous surveys or master data to ensure accuracy. They also removed ‘speeders’ (i.e., individuals who are below 25% of the average completion time), ‘cheaters’ (i.e., Bilendi looked at open-ended responses provided by the individuals: if individuals give illogical or arbitrary answers, or answers that are not related to the question at all, they sorted them out at this point) and ‘straight-liners’ (i.e., Bilendi looked at matrix questions or longer question sequences and carefully checked the variance in the response pattern; if a response is given disproportionately often in context, they also exclude that person).

This study was part of a large multi-thematic survey (with clearly separate questions, e.g., on oral health (assessed by the Oral Health Impact Profile [35]) involving several researchers. We confirm that all instruments (such as PHQ-9) that were considered in advance for this study were used in this analysis and are reported here.

Prior to beginning the survey, all participants gave their written informed consent by agreeing to the online consent form, which is a standard practice for carrying out online surveys. This study received ethics approval from the Local Psychological Ethics Committee at the University Medical Centre Hamburg-Eppendorf (LPEK-0629). This study is in accordance with the Declaration of Helsinki.

### 2.2 Outcomes

Loneliness, objective social isolation, perceived social isolation, and social withdrawal were used as outcomes in this study. The outcomes used are somewhat correlated, but do not measure the same construct [12]. Further details are provided elsewhere [12].

The De Jong Gierveld assessment was used to quantify loneliness [36]. We used the 6-item version with a final score from 0 to 6, whereby higher scores correspond to higher loneliness levels. In our study, Cronbach’s alpha was .81.

The Lubben Social Network Scale (LSNS, 6-item version) measured objective social isolation [37]. It originally ranged from 0 to 30 (whereby higher scores reflect lower objective social isolation levels). However, to make it easier for the reader to interpret (in the sense that all results can be interpreted in the same way: the higher the score, the poorer the rating along the scale), we have reversed the coding: The reversed score also ranges from 0 to 30, with higher scores indicating higher objective social isolation levels. Nevertheless, it is worth noting that the reversed score diverged from standard practice. In this study, Cronbach’s alpha was .87.

Perceived social isolation was quantified using the Bude and Lantermann [13] tool, which has four items. The final score ranges from 1 to 4, whereby higher values indicate higher perceived social isolation levels. In our study, Cronbach’s alpha equaled .91.

Hikikomori symptoms (which reflect social withdrawal, i.e., a voluntary and extended confinement of individuals within their own homes, separate from social interactions [38]) were measured using the German version of the 25-item Hikikomori Questionnaire (HQ-25) [38]. This German version has been recently validated [39]. The final score varies from 0 to 100, whereby higher scores indicate greater social withdrawal (i.e., more hikikomori symptoms). Cronbach’s alpha was .93 in our study.

### 2.3 Key independent variable: type of diet

Based on the tool used to quantify the type of diet in the German Socio-Economic Panel (GSOEP) [40], the key independent variable was measured. The exact wording was: “Do you follow a mainly vegetarian or vegan diet?” The three answer categories were: Yes, vegetarian; Yes, vegan; No, none of the above. The option “no, none of the above” can be classified as individuals following an omnivorous diet (i.e., eating plants and meat/fish). It should be noted that the terms (e.g., “mainly vegetarian”) are vague and are discussed in more detail in the limitations section.

### 2.4 Covariates

Building on prior studies in this field of research, sociodemographic, lifestyle-related, and health-related covariates were chosen for the regression analysis. The following sociodemographic covariates were used: gender (with three categories: men; women; other), age, family situation (with five categories: single; divorced; widowed; living together: married or in partnership; living separately: married or in partnership), educational level according to the CASMIN classification [41] (with three categories: primary education; secondary education; tertiary education), labor participation (with three categories: full-time employment; retired; other), the presence of a migration background (with two categories: no; yes), federal state (covering the 16 federal states of Germany) and religious affiliation (with seven categories: no religious affiliation; Christianity; Judaism; Islam; Buddhism; Hinduism; Other). Due to the low number of cases in some categories, we reduced responses for analytical purposes to four groups: no religious affiliation, Christianity, Islam, or other.

The following lifestyle-related covariates were selected: smoking behavior (with four categories from “never been a smoker” to “daily smoker”), alcohol intake (with six categories from “never” to “daily consumption”), and frequency of sports activities (with five categories from “no sports activity” to “more than 4 hours per week”).

The following health-related covariates were used: self-rated health (a single-item measure with five categories ranging from 1 reflecting very poor self-rated health to 5 reflecting very good self-rated health), and chronic conditions. Guided by the assessment in the German Socio-Economic Panel [42], a count of chronic conditions was generated based on the presence of fourteen specified chronic conditions: Sleep disorder; Thyroid disease; Diabetes; Asthma; Heart disease (also heart failure, cardiac insufficiency); Cancer; Stroke; Migraine; High blood pressure; Dementia; Joint disease (also arthrosis, rheumatism); Chronic back problems; Burnout; Other illness. Moreover, depressive symptoms were used as a mental health covariate. Depressive symptoms were measured using the Patient Health Questionnaire-9 (PHQ-9) [43], with scores ranging from 0 to 27, whereby higher scores indicate more depressive symptoms.

### 2.5 Statistical analysis

The characteristics of the sample, stratified by diet type, are shown first. One-way ANOVAs or Chi-squared tests (as appropriate) were employed for bivariate analysis. Effect sizes (in terms of Cohen’s *d*) were also calculated for the association between type of diet and the outcomes, with *d* = .20 reflecting a small effect, *d* = .50 reflecting a medium effect and *d* = .80 reflecting a large effect [44].

For each dependent variable, we ran five regression models: [1] an unadjusted model (i.e., only ‘type of diet’ was used as an independent variable, i.e., without any other covariates), [2] adding sociodemographic covariates, [3] adding lifestyle-related covariates, [4] adding health-related covariates, [5] and adding the mental health-related covariate. Variance inflation factors (VIFs) were computed for the fully-adjusted model. The mean VIF was 1.48. Many VIFs were considerably less than 1.5, and the highest VIF was 3.11 (tertiary education), indicating that multicollinearity is not a significant concern in our model.

We calculated robust standard errors. In a sensitivity analysis, bootstrapped standard errors (with 1000 reps) were calculated. It is worth noting that the main independent variable (type of diet) was dummy-coded for regression analysis. Specifically, the reference category was “no vegetarian or vegan diet”. Comparison groups were “mainly vegetarian diet” and “mainly vegan diet”. In a sensitivity analysis, outliers in the outcomes were winsorized (highest/lowest 5%).

Effect sizes (i.e., partial eta² values) were calculated for the fully-adjusted model. Such partial eta² values can be classified as follows [44]: 0.01 as “small”, 0.06 as “medium, and 0.14 as “large”, respectively. There were no missing values in the data set because a forced response format was applied in the online survey. Stata 18.0 (Stata Corp., College Station, Texas) was used in our study. The statistical significance was set at p < 0.05.

## 3 Results

### 3.1 Sample characteristics

Our sample is described in Table 1 (stratified by type of diet and for the total sample). Among the total sample, 50.8% of the individuals were female and the average age was 46.9 years, with *SD*: 15.3 years (18–74 years). Overall, 15.4% of the respondents reported following a mainly vegetarian diet and 3.5% of the individuals followed a mainly vegan diet. The remainder of the sample (81.4%) followed an omnivorous diet. The average loneliness score was 3.1 (*SD*: 2.1), the average objective social isolation score was 15.4 (*SD*: 6.1), the average perceived social isolation score was 1.9 (*SD*: 0.8), and the average hikikomori symptoms score was 37.5 (*SD*: 18.1).

**Table 1:** Sample characteristics (stratified by type of diet and total sample).

Variables	Not following a mainly vegetarian or vegan diet	Following a mainly vegetarian diet	Following a mainly vegan diet	Total	P-value
N (%)	4057 (81.1)	768 (15.4)	175 (3.5)	5000 (100.0)	
Gender: N (%)					<0.001
Male	2090 (51.5)	272 (35.4)	89 (50.9)	2451 (49.0)	
Female	1961 (48.3)	493 (64.2)	86 (49.1)	2540 (50.8)	
Other	6 (0.1)	3 (0.4)	0 (0.0)	9 (0.2)	
Age: Mean (SD)	48.4 (14.9)	41.5 (15.5)	34.8 (11.3)	46.9 (15.3)	<0.001
Marital status: N (%)					<0.001
Single	1032 (25.4)	253 (32.9)	48 (27.4)	1333 (26.7)	
Divorced	348 (8.6)	43 (5.6)	12 (6.9)	403 (8.1)	
Widowed	127 (3.1)	24 (3.1)	9 (5.1)	160 (3.2)	
Living together: Married/Partnership	2389 (58.9)	404 (52.6)	100 (57.1)	2893 (57.9)	
Living separated: Married/Partnership	161 (4.0)	44 (5.7)	6 (3.4)	211 (4.2)	
Education: N (%)					<0.001
Primary education	476 (11.7)	40 (5.2)	17 (9.7)	533 (10.7)	
Secondary education	2526 (62.3)	383 (49.9)	78 (44.6)	2987 (59.7)	
Tertiary education	1055 (26.0)	345 (44.9)	80 (45.7)	1480 (29.6)	
Employment status: N (%)					<0.001
Full-time employed	1943 (47.9)	382 (49.7)	93 (53.1)	2418 (48.4)	
Retired	897 (22.1)	95 (12.4)	8 (4.6)	1000 (20.0)	
Other	1217 (30.0)	291 (37.9)	74 (42.3)	1582 (31.6)	
Migration background: N (%)					<0.001
No	3650 (90.0)	649 (84.5)	150 (85.7)	4449 (89.0)	
Yes	407 (10.0)	119 (15.5)	25 (14.3)	551 (11.0)	
Religious affiliation: N (%)					<0.001
Not belonging to any denomination	1923 (47.4)	382 (49.7)	66 (37.7)	2371 (47.4)	
Christianity	2003 (49.4)	324 (42.2)	82 (46.9)	2409 (48.2)	
Islam	85 (2.1)	37 (4.8)	15 (8.6)	137 (2.7)	
Others	46 (1.1)	25 (3.3)	12 (6.9)	83 (1.7)	
Smoking status: N (%)					<0.001
Yes, daily	1019 (25.1)	129 (16.8)	36 (20.6)	1184 (23.7)	
Yes, occasionally	366 (9.0)	99 (12.9)	51 (29.1)	516 (10.3)	
No, not anymore	1145 (28.2)	182 (23.7)	39 (22.3)	1366 (27.3)	
Never smoked before	1527 (37.6)	358 (46.6)	49 (28.0)	1934 (38.7)	
Alcohol consumption: N (%)					<0.001
Daily	215 (5.3)	48 (6.2)	12 (6.9)	275 (5.5)	
Several times a week	847 (20.9)	135 (17.6)	48 (27.4)	1030 (20.6)	
Once a week	752 (18.5)	113 (14.7)	33 (18.9)	898 (18.0)	
1-3 times a month	665 (16.4)	142 (18.5)	21 (12.0)	828 (16.6)	
Less often	900 (22.2)	166 (21.6)	27 (15.4)	1093 (21.9)	
Never	678 (16.7)	164 (21.4)	34 (19.4)	876 (17.5)	
Frequency of sports activity: N (%)					<0.001
No sports activity	1153 (28.4)	93 (12.1)	9 (5.1)	1255 (25.1)	
Less than one hour per week	755 (18.6)	135 (17.6)	27 (15.4)	917 (18.3)	
1-2 hours per week	945 (23.3)	200 (26.0)	57 (32.6)	1202 (24.0)	
2-4 hours per week	672 (16.6)	169 (22.0)	48 (27.4)	889 (17.8)	
More than 4 hours per week	532 (13.1)	171 (22.3)	34 (19.4)	737 (14.7)	
Number of chronic conditions (0–14, whereby higher scores correspond to a higher number of chronic conditions): Mean (SD)	1.5 (1.6)	1.4 (1.5)	1.2 (1.2)	1.5 (1.6)	<0.01
Self-rated health (1 = very poor to 5 = very good): Mean (SD)	3.6 (0.8)	3.9 (0.8)	3.9 (0.8)	3.6 (0.8)	<0.001
Depressive symptoms (PHQ-9; 0–27, whereby higher scores correspond to more depressive symptoms): Mean (SD)	5.3 (5.3)	7.1 (6.0)	8.5 (5.6)	5.7 (5.4)	<0.01
Loneliness (DJG loneliness scale; 0–6, whereby higher scores correspond to higher loneliness levels): Mean (SD)	3.1 (2.1)	3.2 (2.0)	3.7 (1.9)	3.1 (2.1)	<0.01
Objective social isolation (LSNS-6 (reversed); 0–30, whereby higher scores correspond to lower social support/social engagement): Mean (SD)	15.7 (6.1)	14.6 (6.0)	13.7 (6.1)	15.4 (6.1)	<0.001
Perceived social isolation (Bude & Lantermann tool; 1–4, whereby higher scores correspond to more perceived social isolation): Mean (SD)	1.9 (0.8)	2.1 (0.9)	2.3 (0.8)	1.9 (0.8)	<0.001
Hikikomori symptoms (HQ-25-G; 0–100, whereby higher scores correspond to greater social withdrawal): Mean (SD)	36.9 (18.0)	39.5 (18.2)	42.9 (17.3)	37.5 (18.1)	<0.001

Notes: P-Values are based on one-way ANOVAs or Chi²-tests, as appropriate. The federal state (with 16 states) is not shown here due to reasons of readability. Of note, the federal state was significantly associated with the type of diet (p = 0.02).

According to bivariate analysis, the type of diet was significantly associated with all other variables. The effect sizes (Cohen’s *d*) for the association between type of diet and outcomes are shown in Table 2. There were small differences between individuals following a mainly vegetarian diet and individuals following a mainly vegan diet, with the latter group reporting poorer outcomes (except for objective social isolation). There were also small differences regarding the outcomes between individuals following a mainly vegetarian diet and individuals following an omnivorous diet, with the former group reporting poorer outcomes (again, except for objective social isolation). Moreover, small to medium (perceived social isolation) differences were present between individuals following a mainly vegan diet and individuals following an omnivorous diet, with the former group reporting poorer outcomes (again, except for objective social isolation).

**Table 2:** Effect sizes (Cohen’s *d*) for the association between type of diet and social outcomes.

	Loneliness	Objective social isolation	Perceived social isolation	Social withdrawal
Vegetarian vs. vegan	−.24	.15	−.29	−.19
Vegetarian vs. omnivore	.05	−.18	.25	.14
Vegan vs. omnivore	.27	−.33	.55	.34

Notes: Please note that Cohen’s *d* refers to unadjusted effect sizes. A positive sign means that the former group has higher mean values than the latter group. A negative sign means that the former group has lower mean values than the latter group. Here are some examples of interpretations: *d* = .27 (vegan vs. omnivore in loneliness) means that individuals following a mainly vegan diet have higher loneliness scores than individuals following a mainly omnivorous diet; *d* = −.24 (vegetarian vs. vegan in loneliness) means that individuals following a mainly vegetarian diet have lower loneliness scores than individuals following a mainly vegan diet.

### 3.2 Regression analysis

Unadjusted and adjusted results of linear regressions are shown in Table 3 (regressions displaying all covariates are shown in S1 Table). When adjusting for sociodemographic lifestyle-related and health-related factors, individuals following a mainly vegetarian diet had significantly higher levels of perceived social isolation (β = 0.15, p < .001) and social withdrawal (β = 2.77, p < .001) compared to individuals following an omnivorous diet. However, these significant associations disappeared when additionally adjusting for mental health-related covariates (i.e., depressive symptoms).

**Table 3:** Association of type of diet with loneliness, social isolation and social withdrawal. Results based on linear regressions.

Independent variables	Loneliness					Objective social isolation					Perceived social isolation					Social withdrawal				
Type of diet:																				
- Mainly vegetarian diet	0.11	0.04	0.08	0.14+	−0.09	−1.11***	−0.45*	−0.25	−0.08	−0.36+	0.21***	0.11**	0.12***	0.15***	0.02	2.59***	1.87**	2.28**	2.77***	0.56
	(−0.05 - 0.26) [0.02]	(−0.12 - 0.20) [0.007]	(−0.08 - 0.24) [0.01]	(−0.01 - 0.30) [0.02]	(−0.23 - 0.05) [-0.02]	(−1.57 - −0.65) [-0.07]	(−0.89 - −0.00) [-0.03]	(−0.68 - 0.18) [-0.01]	(−0.50 - 0.34) [-0.005]	(−0.78 - 0.06) [-0.02]	(0.14 - 0.27) [0.09]	(0.04 - 0.17) [0.05]	(0.06 - 0.19) [0.05]	(0.09 - 0.21) [0.06]	(−0.03 - 0.07) [0.01]	(1.19 - 3.99) [0.05]	(0.46 - 3.27) [0.04]	(0.90 - 3.66) [0.05]	(1.42 - 4.12) [0.06]	(−0.66 - 1.77) [0.01]
- Mainly vegan diet	0.58***	0.47**	0.46**	0.48**	0.20	−2.00***	−0.48	−0.08	−0.02	−0.36	0.46***	0.26***	0.24***	0.25***	0.10*	6.03***	4.35***	4.75***	4.87***	2.24*
	(0.29 - 0.86) [0.05]	(0.18 - 0.75) [0.04]	(0.17 - 0.75) [0.04]	(0.19 - 0.77) [0.04]	(−0.07 - 0.48) [0.02]	(−2.92 - −1.08) [-0.06]	(−1.35 - 0.38) [-0.01]	(−0.93 - 0.77) [-0.002]	(−0.87 - 0.82) [-0.001]	(−1.21 - 0.49) [-0.01]	(0.34 - 0.58) [0.10]	(0.14 - 0.38) [0.06]	(0.12 - 0.37) [0.05]	(0.13 - 0.37) [0.05]	(0.002 - 0.19) [0.02]	(3.42 - 8.64) [0.06]	(1.77 - 6.93) [0.04]	(2.17 - 7.33) [0.05]	(2.27 - 7.46) [0.05]	(0.04 - 4.44) [0.02]
Sociodemographic covariates		✔	✔	✔	✔		✔	✔	✔	✔		✔	✔	✔	✔		✔	✔	✔	✔
Lifestyle-related covariates			✔	✔	✔			✔	✔	✔			✔	✔	✔			✔	✔	✔
Health-related covariates				✔	✔				✔	✔				✔	✔				✔	✔
Mental health-related covariates					✔					✔					✔					✔
Constant	3.10***	4.46***	4.93***	7.43***	4.43***	15.66***	15.44***	18.87***	24.32***	20.65***	1.88***	2.79***	2.89***	3.97***	2.33***	36.89***	56.05***	64.76***	82.86***	54.17***
	(3.03 - 3.16)	(4.11 - 4.81)	(4.55 - 5.32)	(6.97 - 7.90)	(3.94 - 4.93)	(15.47 - 15.85)	(14.41 - 16.47)	(17.77 - 19.98)	(22.93 - 25.70)	(19.12 - 22.18)	(1.86 - 1.91)	(2.65 - 2.93)	(2.73 - 3.05)	(3.78 - 4.15)	(2.15 - 2.50)	(36.33 - 37.44)	(53.12 - 58.98)	(61.57 - 67.96)	(78.91 - 86.80)	(50.07 - 58.27)
R²	0.00	0.07	0.10	0.17	0.29	0.01	0.13	0.19	0.22	0.24	0.02	0.13	0.15	0.24	0.45	0.01	0.10	0.15	0.20	0.33
Adjusted R²	0.00	0.07	0.09	0.17	0.28	0.01	0.13	0.18	0.21	0.23	0.02	0.12	0.14	0.24	0.44	0.01	0.10	0.14	0.19	0.33

In all of the regression models presented, “No vegetarian or vegan diet” served as the reference category; The number of observations was n = 5,000 in all models; Unstandardized beta-coefficients; 95% confidence intervals in parentheses; standardized beta-coefficients in brackets; *** p < 0.001, ** p < 0.01, * p < 0.05, + p < 0.10; sociodemographic covariates: gender, age, marital status, federal state, education, employment situation, migration background and religious affiliation; lifestyle-related covariates: frequency of sports activities, smoking behavior, and alcohol consumption; health-related covariates: self-rated health, and count of chronic conditions; mental health-related covariates: depressive symptoms.

Additionally, vegan eaters feel lonelier (β = 0.48, p < .01) compared to individuals following an omnivorous diet when adjusting for sociodemographic, lifestyle-related and health-related covariates. Similarly, this significant association disappeared when adjusting for mental health-related covariates. However, vegan eaters feel more isolated (β = 0.10, p < .05) and withdraw more from society (β = 2.24, p < .05), compared to individuals following an omnivorous diet, even in the fully-adjusted model. In a sensitivity analysis (see S2 Table), bootstrapped standard errors were calculated. Such findings remained virtually the same compared to the results presented in Table 3. In a further sensitivity analysis, outliers in the outcomes were winsorized. However, findings remained nearly the same (in the fully adjusted model: mainly vegan, with perceived social isolation as outcome: β = .10, p < .05; mainly vegan, with social withdrawal as outcome: β = 2.13, p < .05).

Of note, the partial eta² values were consistently very small for the type of diet (i.e., smaller than 0.01). The type of diet explains only a negligible proportion of variance in all outcomes. It should be noted that the inclusion of depressive symptoms explains a great deal of variance.

## 4 Discussion

Using data from the general adult German population, the aim of this study was to investigate the association of type of diet with loneliness, social isolation and social withdrawal. This study found that those following a mainly vegetarian diet had significantly higher levels of perceived social isolation and social withdrawal compared to those following an omnivorous diet, when adjusting for sociodemographic and lifestyle-related factors. However, these associations were non-significant when additionally adjusting for mental health-related covariates (i.e., depressive symptoms). Notably, individuals following a mainly vegetarian diet did not have significantly different levels of loneliness (i.e., not significant in any model) and objective social isolation (when adjusting for sociodemographic and lifestyle-related covariates) compared to those following an omnivorous diet. A mainly vegan diet (compared to a mainly omnivorous diet) is significantly associated with higher levels of perceived social isolation and social withdrawal, even in the fully-adjusted model. Although some associations were statistically significant, the variance explained by diet type was minimal (R² close to zero and partial eta² < 0.01), suggesting limited practical relevance. In the following discussion, it is essential to state this point with utmost clarity to prevent any risk of misinterpretation of the results. This is particularly important given the large sample size, which increases the likelihood of obtaining statistically significant results.

A few qualitative studies suggest that there may be an association between being vegetarian/vegan and feeling lonely [15,23]. This first *quantitative* study adds considerably to this sparse knowledge base. This present study also incorporates outcomes of social isolation and social withdrawal. Overall, our study provides a more nuanced and detailed perspective than one might initially anticipate from the aforementioned qualitative studies.

The (very small) association between following a mainly vegan diet and higher levels of perceived social isolation and social withdrawal may be explained by several factors. Generally, vegans could feel that their dietary habits are not taken seriously in German society and may feel discriminated against [32]. The (very small) associations between following a mainly vegan diet and higher levels of perceived social isolation and social withdrawal may be stronger in regions where vegan diets have a rather low level of acceptance (possibly in more rural areas with fewer vegan options in supermarkets and restaurants). Moreover, as described in the introduction section, our results can also be interpreted within the framework of stigma and minority stress theory. Another explanation may be that dietary practices are very important for their identity among vegans compared to vegetarians and particularly when compared to individuals with omnivorous diets [45]. This self-identity is important for embitterment among vegans – as previous research has shown [32]. Embitterment eventually can contribute to loneliness, isolation and social withdrawal [33]. However, we would like to emphasize once again that the variance explained by diet type was minimal, which suggests a limited practical significance.

Some strengths and limitations of our work are worth noting. This is the first quantitative study investigating the association of type of diet with social isolation, loneliness and social withdrawal. Data were taken from a large sample, ensuring representativeness for the adult population in Germany in terms of gender, age and federal state. However, our quota-based survey may not guarantee representativeness with respect to other characteristics (e.g., citizenship). Therefore, future research based on random samples is encouraged. Valid tools were used to quantify loneliness, social isolation and social withdrawal. Moreover, several covariates were included in the regression analysis. The item used to quantify the type of diet has a high face validity and was also used in other large household panels [40]. Nevertheless, the type of diet could be quantified in further detail in future studies. It is also a subjective assessment of what exactly is meant by ‘mainly’ vegetarian/vegan. For example, while one person may describe themselves as mainly vegetarian and eat meat several times a week, another person might not describe themselves as mainly vegetarian and eat meat only once a month. It is worth noting that the proportion of vegans in our study is similar to figures reported in a previous study based on the German adult population [1]. This may demonstrate that most individuals tend to use stricter definitions of veganism. Notably, the proportion of vegetarians in our study is higher compared to previous research [46]. Additionally, future research could focus on the association of dietary identity and social disconnectedness outcomes. It should be noted that no information was collected on factors such as motivation, duration, or strength of adherence to diet. Such factors may bias our present findings.

Moreover, clarifying the directionality between the variables of interest is challenging due to the cross-sectional design of our study. In this regard, we would like to emphasize that we have demonstrated *associations* between type of diet and social disconnectedness outcomes. People who, for example, withdraw socially or develop a greater sense of loneliness over time may change their lifestyle [47,48]. For example, they may change their physical activity [49]. They may also adjust their type of diet [50,51]. Therefore, we recommend future longitudinal studies in order to analyze this complex relationship in greater depth. From a historical perspective, it should be noted that Germany is a meat-centric country. Comparisons with other countries (such as India – historically, a country with a diet that is very low in meat) would be of interest. Moreover, it is also questionable to what extent our current results for Germany can be applied to cultures or countries with different eating habits, where, for example, eating alone may be more socially acceptable (such as Japan). Previous research also showed that the personality factors of openness/intellect and agreeableness are associated with higher levels of vegetarianism/veganism [52] (see also: [53]). Future research could also further explore the role of personality in the association of the type of diet with social disconnectedness outcomes. Using data from an online sample may also introduce some selection bias. This may be particularly the case for people who are driven by their beliefs or those who are very health-conscious. Furthermore, other factors may be of relevance here, such as eating disorders [54]. Due to reasons of data availability, such factors were not taken into account in the present study. However, such factors should be further explored in future studies.

## 5 Conclusion

In conclusion, following a mainly vegan diet was associated with perceived social isolation and social withdrawal even when adjusting for sociodemographic, lifestyle-related, health-related, and mental health-related covariates. However, diet type accounted for only a negligible proportion of the variance. This indicates restricted practical significance.

Guided by the minority stress theory and stigma theory [25,26], one might assume that vegetarians and vegans experience stigmatization due to a lack of acceptance and social prejudice. Thus, future research in other countries where vegetarian or vegan diets are widespread, such as India or Switzerland, or limited, such as China or Japan, may be beneficial [55]. This is also of interest because in some countries, such as India, there is a strong association between a vegetarian diet and religious affiliation, whereas following a vegetarian or vegan diet can be mainly explained by ecological or health reasons in Germany [2].

Eating occasions could be analyzed in detail in future studies (e.g., differentiating between working lunches, breakfasts, visits to restaurants with friends and family and others). A distinction could also be made between other types of diets such as pescatarians, flexitarians, fruitarians or individuals following a gluten-free or lactose-free diet. Future research could also investigate whether there are differences between urban and rural areas (in the context of food culture).

Overall, our study provided the first quantitative evidence of a very small association between certain plant-based diets and loneliness, social isolation and social withdrawal in Germany. We hope that our work may contribute to further research in this under-researched area.

## Supporting information

S1 Table Association of type of diet with loneliness, social isolation and social withdrawal. Results based on multiple linear regressions (all covariates are shown).
(DOC)

S2 Table Association of type of diet with loneliness, social isolation and social withdrawal. Results based on multiple linear regressions (with bootstrapped standard errors).
(DOC)
