# Supplementary material for: Association of mainly vegetarian and vegan diets with loneliness, social isolation and social withdrawal in a German population survey
Source: PLoS One. 2026 Jul 15;21(7):e0353869. doi: 10.1371/journal.pone.0353869 (PMC13372175; doi:10.1371/journal.pone.0353869)
Supplement: S1 Table — Results based on multiple linear regressions (all covariates are shown). (DOC) [file pone.0353869.s001.doc]

S1 Table. Association of type of diet with loneliness, social isolation and social withdrawal. Results based on multiple linear regressions (all covariates are shown)
Independent variables	Loneliness	Objective social isolation	Perceived social isolation	Social withdrawal	
																	
																	
Type of diet: 																	
																	
- No vegetarian or vegan diet	Reference category	Reference category	Reference category	Reference category	Reference category	Reference category	Reference category	Reference category	Reference category	Reference category	Reference category	Reference category	Reference category	Reference category	Reference category	Reference category	
																	
- Mainly vegetarian diet	0.04	0.08	0.14+	-0.09	-0.45*	-0.25	-0.08	-0.36+	0.11**	0.12***	0.15***	0.02	1.87**	2.28**	2.77***	0.56	
	(-0.12 - 0.20)	(-0.08 - 0.24)	(-0.01 - 0.30)	(-0.23 - 0.05)	(-0.89 - -0.00)	(-0.68 - 0.18)	(-0.50 - 0.34)	(-0.78 - 0.06)	(0.04 - 0.17)	(0.06 - 0.19)	(0.09 - 0.21)	(-0.03 - 0.07)	(0.46 - 3.27)	(0.90 - 3.66)	(1.42 - 4.12)	(-0.66 - 1.77)	
- Mainly vegan diet	0.47**	0.46**	0.48**	0.20	-0.48	-0.08	-0.02	-0.36	0.26***	0.24***	0.25***	0.10*	4.35***	4.75***	4.87***	2.24*	
	(0.18 - 0.75)	(0.17 - 0.75)	(0.19 - 0.77)	(-0.07 - 0.48)	(-1.35 - 0.38)	(-0.93 - 0.77)	(-0.87 - 0.82)	(-1.21 - 0.49)	(0.14 - 0.38)	(0.12 - 0.37)	(0.13 - 0.37)	(0.00 - 0.19)	(1.77 - 6.93)	(2.17 - 7.33)	(2.27 - 7.46)	(0.04 - 4.44)	
																	
Gender: 																	
																	
- Men	Reference category	Reference category	Reference category	Reference category	Reference category	Reference category	Reference category	Reference category	Reference category	Reference category	Reference category	Reference category	Reference category	Reference category	Reference category	Reference category	
																	
- Women	-0.03	-0.05	-0.06	-0.15**	-0.05	-0.35*	-0.34*	-0.45**	-0.01	-0.00	-0.01	-0.06**	-2.30***	-2.44***	-2.52***	-3.37***	
	(-0.15 - 0.08)	(-0.17 - 0.07)	(-0.18 - 0.05)	(-0.26 - -0.04)	(-0.38 - 0.29)	(-0.69 - -0.01)	(-0.68 - -0.01)	(-0.78 - -0.12)	(-0.06 - 0.03)	(-0.05 - 0.05)	(-0.05 - 0.04)	(-0.10 - -0.02)	(-3.32 - -1.28)	(-3.47 - -1.41)	(-3.52 - -1.51)	(-4.29 - -2.45)	
- Other	-0.22	-0.33	-0.71	-1.11*	0.74	-0.01	-0.75	-1.25	0.38	0.37	0.21	-0.02	2.04	0.77	-1.93	-5.81	
	(-1.43 - 0.98)	(-1.48 - 0.82)	(-1.81 - 0.39)	(-2.20 - -0.03)	(-1.94 - 3.42)	(-2.96 - 2.95)	(-4.03 - 2.52)	(-4.64 - 2.14)	(-0.15 - 0.91)	(-0.16 - 0.90)	(-0.24 - 0.65)	(-0.42 - 0.39)	(-7.79 - 11.87)	(-10.07 - 11.61)	(-11.80 - 7.94)	(-13.94 - 2.33)	
																	
Age	-0.01***	-0.01***	-0.02***	-0.00	0.08***	0.07***	0.05***	0.08***	-0.02***	-0.02***	-0.02***	-0.01***	-0.20***	-0.22***	-0.28***	-0.06**	
	(-0.02 - -0.01)	(-0.02 - -0.01)	(-0.03 - -0.02)	(-0.01 - 0.00)	(0.06 - 0.09)	(0.05 - 0.08)	(0.04 - 0.07)	(0.07 - 0.10)	(-0.02 - -0.01)	(-0.02 - -0.02)	(-0.02 - -0.02)	(-0.01 - -0.01)	(-0.24 - -0.15)	(-0.26 - -0.18)	(-0.33 - -0.24)	(-0.10 - -0.02)	
																	
Marital status: 																	
																	
- Single	Reference category	Reference category	Reference category	Reference category	Reference category	Reference category	Reference category	Reference category	Reference category	Reference category	Reference category	Reference category	Reference category	Reference category	Reference category	Reference category	
																	
- Divorced	-0.09	-0.13	-0.13	-0.12	-0.23	-0.19	-0.15	-0.14	-0.00	-0.02	-0.02	-0.01	-1.87+	-1.98*	-1.95*	-1.87*	
	(-0.33 - 0.15)	(-0.37 - 0.11)	(-0.35 - 0.10)	(-0.32 - 0.09)	(-0.87 - 0.42)	(-0.82 - 0.44)	(-0.78 - 0.47)	(-0.76 - 0.47)	(-0.10 - 0.10)	(-0.11 - 0.08)	(-0.11 - 0.07)	(-0.09 - 0.06)	(-3.91 - 0.17)	(-3.95 - -0.01)	(-3.85 - -0.04)	(-3.61 - -0.12)	
- Widowed	-0.48**	-0.53**	-0.35*	-0.38*	-1.87***	-1.79***	-1.40**	-1.43**	-0.15*	-0.17*	-0.09	-0.11*	-2.94+	-3.28*	-1.95	-2.25+	
	(-0.83 - -0.14)	(-0.88 - -0.19)	(-0.67 - -0.02)	(-0.68 - -0.08)	(-2.84 - -0.90)	(-2.72 - -0.85)	(-2.31 - -0.48)	(-2.35 - -0.52)	(-0.28 - -0.01)	(-0.31 - -0.04)	(-0.22 - 0.03)	(-0.21 - -0.01)	(-6.00 - 0.12)	(-6.23 - -0.33)	(-4.81 - 0.91)	(-4.88 - 0.38)	
- Living together: married or in partnership	-0.83***	-0.81***	-0.67***	-0.56***	-2.45***	-2.14***	-1.85***	-1.72***	-0.24***	-0.24***	-0.19***	-0.13***	-8.04***	-7.49***	-6.51***	-5.48***	
	(-0.96 - -0.69)	(-0.94 - -0.67)	(-0.80 - -0.54)	(-0.69 - -0.44)	(-2.85 - -2.05)	(-2.53 - -1.76)	(-2.23 - -1.47)	(-2.10 - -1.34)	(-0.29 - -0.18)	(-0.30 - -0.19)	(-0.24 - -0.13)	(-0.17 - -0.08)	(-9.19 - -6.89)	(-8.61 - -6.36)	(-7.61 - -5.42)	(-6.49 - -4.47)	
- Living separately: married or in partnership)	-0.98***	-0.95***	-0.87***	-0.84***	-1.03*	-0.79*	-0.60	-0.56	-0.25***	-0.25***	-0.21***	-0.19***	-8.42***	-7.91***	-7.29***	-6.97***	
	(-1.28 - -0.67)	(-1.26 - -0.65)	(-1.15 - -0.58)	(-1.11 - -0.57)	(-1.82 - -0.24)	(-1.54 - -0.04)	(-1.34 - 0.14)	(-1.29 - 0.17)	(-0.36 - -0.13)	(-0.36 - -0.13)	(-0.32 - -0.10)	(-0.28 - -0.10)	(-10.95 - -5.89)	(-10.38 - -5.44)	(-9.67 - -4.90)	(-9.12 - -4.82)	
																	
Education: 																	
																	
- Primay education	Reference category	Reference category	Reference category	Reference category	Reference category	Reference category	Reference category	Reference category	Reference category	Reference category	Reference category	Reference category	Reference category	Reference category	Reference category	Reference category	
																	
- Secondary education	-0.25*	-0.16+	-0.12	-0.12	-1.16***	-0.73*	-0.65*	-0.66*	-0.06	-0.04	-0.02	-0.03	-2.95***	-1.96*	-1.66*	-1.69*	
	(-0.44 - -0.05)	(-0.36 - 0.03)	(-0.31 - 0.06)	(-0.29 - 0.04)	(-1.74 - -0.58)	(-1.30 - -0.16)	(-1.20 - -0.10)	(-1.20 - -0.11)	(-0.14 - 0.02)	(-0.12 - 0.04)	(-0.10 - 0.05)	(-0.09 - 0.04)	(-4.58 - -1.31)	(-3.57 - -0.35)	(-3.20 - -0.11)	(-3.12 - -0.26)	
- Tertiary education	-0.59***	-0.41***	-0.29**	-0.27**	-2.89***	-1.99***	-1.78***	-1.76***	-0.15***	-0.11*	-0.06	-0.05	-5.84***	-3.88***	-3.08***	-2.91***	
	(-0.80 - -0.38)	(-0.62 - -0.19)	(-0.50 - -0.09)	(-0.47 - -0.08)	(-3.53 - -2.26)	(-2.63 - -1.35)	(-2.41 - -1.16)	(-2.38 - -1.14)	(-0.24 - -0.07)	(-0.20 - -0.02)	(-0.15 - 0.02)	(-0.12 - 0.02)	(-7.65 - -4.03)	(-5.70 - -2.06)	(-4.83 - -1.33)	(-4.52 - -1.30)	
																	
Labour participation:																	
																	
- Full-time employed	Reference category	Reference category	Reference category	Reference category	Reference category	Reference category	Reference category	Reference category	Reference category	Reference category	Reference category	Reference category	Reference category	Reference category	Reference category	Reference category	
																	
- Retired	0.16+	0.08	-0.18*	-0.23**	0.55*	0.03	-0.37	-0.43+	0.26***	0.25***	0.13***	0.11***	4.86***	3.71***	1.90*	1.44*	
	(-0.03 - 0.35)	(-0.10 - 0.27)	(-0.36 - -0.00)	(-0.40 - -0.06)	(0.01 - 1.08)	(-0.49 - 0.55)	(-0.89 - 0.14)	(-0.94 - 0.08)	(0.19 - 0.34)	(0.18 - 0.33)	(0.06 - 0.20)	(0.05 - 0.16)	(3.27 - 6.45)	(2.16 - 5.26)	(0.38 - 3.41)	(0.04 - 2.83)	
- Other	0.20**	0.14*	0.00	0.00	1.18***	0.80***	0.56**	0.56**	0.13***	0.13***	0.07*	0.06**	2.03***	1.27*	0.28	0.26	
	(0.06 - 0.33)	(0.01 - 0.28)	(-0.13 - 0.14)	(-0.12 - 0.13)	(0.79 - 1.56)	(0.43 - 1.18)	(0.19 - 0.93)	(0.19 - 0.92)	(0.08 - 0.19)	(0.08 - 0.18)	(0.01 - 0.12)	(0.02 - 0.11)	(0.85 - 3.20)	(0.11 - 2.42)	(-0.85 - 1.40)	(-0.77 - 1.28)	
																	
Migration background:																	
																	
- Not having a migration background	Reference category	Reference category	Reference category	Reference category	Reference category	Reference category	Reference category	Reference category	Reference category	Reference category	Reference category	Reference category	Reference category	Reference category	Reference category	Reference category	
																	
- Having a migration background	0.54***	0.52***	0.51***	0.34***	0.07	0.08	0.09	-0.12	0.20***	0.19***	0.19***	0.09**	2.89***	2.81***	2.76***	1.11	
	(0.36 - 0.73)	(0.34 - 0.70)	(0.34 - 0.69)	(0.18 - 0.51)	(-0.48 - 0.63)	(-0.46 - 0.62)	(-0.44 - 0.63)	(-0.65 - 0.41)	(0.13 - 0.28)	(0.12 - 0.27)	(0.11 - 0.26)	(0.03 - 0.15)	(1.24 - 4.54)	(1.20 - 4.42)	(1.18 - 4.34)	(-0.31 - 2.52)	
																	
Religious affiliation:																	
																	
- No religious affiliation	Reference category	Reference category	Reference category	Reference category	Reference category	Reference category	Reference category	Reference category	Reference category	Reference category	Reference category	Reference category	Reference category	Reference category	Reference category	Reference category	
																	
- Christianity	-0.07	-0.05	-0.07	-0.11*	-1.44***	-1.32***	-1.35***	-1.39***	0.00	0.00	-0.01	-0.03	-2.53***	-2.25***	-2.38***	-2.74***	
	(-0.19 - 0.05)	(-0.17 - 0.07)	(-0.18 - 0.04)	(-0.21 - -0.00)	(-1.78 - -1.10)	(-1.65 - -0.99)	(-1.67 - -1.02)	(-1.71 - -1.07)	(-0.05 - 0.05)	(-0.04 - 0.05)	(-0.05 - 0.04)	(-0.07 - 0.01)	(-3.55 - -1.50)	(-3.26 - -1.25)	(-3.36 - -1.41)	(-3.63 - -1.85)	
- Islam	-0.13	-0.24	-0.03	-0.21	-1.85***	-2.00***	-1.59**	-1.80***	0.08	0.03	0.13	0.03	-0.18	-1.43	0.07	-1.58	
	(-0.47 - 0.21)	(-0.59 - 0.10)	(-0.37 - 0.30)	(-0.53 - 0.12)	(-2.93 - -0.78)	(-3.07 - -0.93)	(-2.61 - -0.56)	(-2.86 - -0.74)	(-0.08 - 0.25)	(-0.13 - 0.19)	(-0.03 - 0.28)	(-0.09 - 0.16)	(-3.69 - 3.33)	(-4.95 - 2.10)	(-3.42 - 3.55)	(-4.76 - 1.60)	
- Other	-0.01	0.02	-0.12	-0.21	-0.61	-0.43	-0.59	-0.69	0.12	0.12	0.05	0.01	0.89	0.99	0.04	-0.77	
	(-0.40 - 0.38)	(-0.35 - 0.39)	(-0.48 - 0.24)	(-0.57 - 0.15)	(-1.88 - 0.65)	(-1.61 - 0.75)	(-1.72 - 0.54)	(-1.86 - 0.48)	(-0.05 - 0.29)	(-0.05 - 0.29)	(-0.11 - 0.21)	(-0.13 - 0.14)	(-2.59 - 4.36)	(-2.38 - 4.36)	(-3.28 - 3.36)	(-3.67 - 2.14)	
																	
Smoking status:																	
																	
- Never been a smoker	Reference category	Reference category	Reference category	Reference category	Reference category	Reference category	Reference category	Reference category	Reference category	Reference category	Reference category	Reference category	Reference category	Reference category	Reference category	Reference category	
																	
- Yes, daily		0.01	-0.06	-0.14*		-0.26	-0.33	-0.43*		-0.01	-0.04	-0.09***		-1.33*	-1.76**	-2.59***	
		(-0.14 - 0.16)	(-0.20 - 0.09)	(-0.28 - -0.01)		(-0.69 - 0.16)	(-0.74 - 0.09)	(-0.85 - -0.02)		(-0.07 - 0.05)	(-0.10 - 0.01)	(-0.14 - -0.04)		(-2.63 - -0.04)	(-3.01 - -0.51)	(-3.75 - -1.44)	
- Yes, occassionally		0.42***	0.39***	0.06		-0.18	-0.17	-0.56*		0.18***	0.16***	-0.01		2.48**	2.25*	-0.83	
		(0.23 - 0.62)	(0.20 - 0.58)	(-0.11 - 0.24)		(-0.73 - 0.38)	(-0.72 - 0.38)	(-1.11 - -0.01)		(0.10 - 0.26)	(0.09 - 0.24)	(-0.08 - 0.05)		(0.74 - 4.22)	(0.54 - 3.97)	(-2.35 - 0.69)	
- No, not anymore		0.18*	0.09	0.06		0.22	0.12	0.09		0.05+	0.00	-0.01		0.19	-0.42	-0.67	
		(0.03 - 0.32)	(-0.05 - 0.23)	(-0.07 - 0.19)		(-0.18 - 0.61)	(-0.27 - 0.52)	(-0.29 - 0.48)		(-0.01 - 0.10)	(-0.05 - 0.06)	(-0.06 - 0.04)		(-1.00 - 1.39)	(-1.59 - 0.75)	(-1.74 - 0.40)	
																	
Alcohol intake: 																	
																	
- Never	Reference category	Reference category	Reference category	Reference category	Reference category	Reference category	Reference category	Reference category	Reference category	Reference category	Reference category	Reference category	Reference category	Reference category	Reference category	Reference category	
																	
- Daily		0.11	0.19	-0.11		-2.38***	-2.16***	-2.54***		0.19**	0.22***	0.06		-0.66	-0.04	-2.95**	
		(-0.16 - 0.38)	(-0.06 - 0.45)	(-0.35 - 0.13)		(-3.19 - -1.57)	(-2.94 - -1.39)	(-3.32 - -1.75)		(0.08 - 0.30)	(0.12 - 0.33)	(-0.03 - 0.14)		(-3.02 - 1.69)	(-2.30 - 2.22)	(-5.05 - -0.85)	
- Several times a week		-0.30**	-0.13	-0.22*		-2.54***	-2.22***	-2.33***		0.01	0.09*	0.04		-4.90***	-3.70***	-4.59***	
		(-0.49 - -0.11)	(-0.31 - 0.05)	(-0.39 - -0.05)		(-3.09 - -1.99)	(-2.76 - -1.68)	(-2.87 - -1.80)		(-0.06 - 0.09)	(0.02 - 0.16)	(-0.02 - 0.10)		(-6.54 - -3.26)	(-5.29 - -2.11)	(-6.05 - -3.13)	
- Once a week		-0.48***	-0.31**	-0.30***		-2.77***	-2.46***	-2.45***		-0.07+	0.01	0.01		-6.67***	-5.45***	-5.33***	
		(-0.68 - -0.29)	(-0.50 - -0.12)	(-0.47 - -0.12)		(-3.30 - -2.23)	(-2.99 - -1.94)	(-2.96 - -1.93)		(-0.15 - 0.00)	(-0.07 - 0.08)	(-0.05 - 0.07)		(-8.32 - -5.02)	(-7.04 - -3.86)	(-6.79 - -3.86)	
- 1-3 times a month		-0.40***	-0.25**	-0.23*		-2.28***	-2.03***	-1.99***		-0.10*	-0.04	-0.02		-6.70***	-5.68***	-5.42***	
		(-0.59 - -0.20)	(-0.44 - -0.07)	(-0.40 - -0.05)		(-2.82 - -1.74)	(-2.56 - -1.49)	(-2.52 - -1.47)		(-0.18 - -0.02)	(-0.11 - 0.04)	(-0.09 - 0.04)		(-8.34 - -5.05)	(-7.27 - -4.08)	(-6.89 - -3.96)	
- Less often		-0.11	-0.04	-0.02		-1.10***	-0.94***	-0.91***		-0.02	0.01	0.02		-3.29***	-2.80***	-2.60***	
		(-0.29 - 0.07)	(-0.22 - 0.13)	(-0.19 - 0.14)		(-1.61 - -0.58)	(-1.44 - -0.43)	(-1.41 - -0.41)		(-0.09 - 0.06)	(-0.06 - 0.08)	(-0.04 - 0.08)		(-4.85 - -1.72)	(-4.31 - -1.29)	(-3.99 - -1.20)	
																	
Frequency of sports activites:																	
																	
- No sports activity	Reference category	Reference category	Reference category	Reference category	Reference category	Reference category	Reference category	Reference category	Reference category	Reference category	Reference category	Reference category	Reference category	Reference category	Reference category	Reference category	
																	
- Less than 1 hour a week		-0.09	-0.03	-0.10		-1.46***	-1.33***	-1.41***		-0.01	0.01	-0.02		-3.93***	-3.49***	-4.17***	
		(-0.27 - 0.08)	(-0.20 - 0.14)	(-0.26 - 0.05)		(-1.95 - -0.97)	(-1.81 - -0.85)	(-1.89 - -0.94)		(-0.08 - 0.06)	(-0.05 - 0.08)	(-0.08 - 0.03)		(-5.37 - -2.49)	(-4.89 - -2.09)	(-5.46 - -2.88)	
- 1 to 2 hours a week		-0.30***	-0.14+	-0.16*		-1.80***	-1.48***	-1.50***		-0.06+	0.02	0.00		-4.88***	-3.71***	-3.91***	
		(-0.47 - -0.14)	(-0.30 - 0.02)	(-0.31 - -0.01)		(-2.26 - -1.33)	(-1.93 - -1.02)	(-1.95 - -1.05)		(-0.12 - 0.01)	(-0.05 - 0.08)	(-0.05 - 0.06)		(-6.28 - -3.48)	(-5.08 - -2.33)	(-5.16 - -2.66)	
- 2 to 4 hours a week		-0.50***	-0.27**	-0.25**		-2.57***	-2.08***	-2.06***		-0.15***	-0.05	-0.04		-7.19***	-5.51***	-5.38***	
		(-0.68 - -0.32)	(-0.44 - -0.09)	(-0.42 - -0.09)		(-3.06 - -2.08)	(-2.56 - -1.59)	(-2.54 - -1.58)		(-0.22 - -0.08)	(-0.11 - 0.02)	(-0.10 - 0.02)		(-8.71 - -5.68)	(-7.01 - -4.02)	(-6.77 - -4.00)	
- More than 4 hours a week		-0.80***	-0.41***	-0.38***		-3.13***	-2.36***	-2.32***		-0.29***	-0.11**	-0.10**		-8.82***	-6.05***	-5.73***	
		(-0.99 - -0.61)	(-0.60 - -0.23)	(-0.55 - -0.20)		(-3.68 - -2.59)	(-2.91 - -1.81)	(-2.86 - -1.78)		(-0.36 - -0.21)	(-0.19 - -0.04)	(-0.16 - -0.03)		(-10.47 - -7.16)	(-7.69 - -4.40)	(-7.24 - -4.21)	
																	
Chronic conditions			0.10***	-0.02			-0.06	-0.20***			0.06***	-0.01			0.58**	-0.59***	
			(0.06 - 0.14)	(-0.06 - 0.01)			(-0.17 - 0.06)	(-0.32 - -0.09)			(0.04 - 0.07)	(-0.02 - 0.00)			(0.23 - 0.93)	(-0.91 - -0.26)	
																	
Self-rated health			-0.67***	-0.29***			-1.46***	-1.00***			-0.29***	-0.08***			-4.84***	-1.22***	
			(-0.75 - -0.59)	(-0.37 - -0.21)			(-1.69 - -1.23)	(-1.24 - -0.75)			(-0.32 - -0.26)	(-0.11 - -0.05)			(-5.53 - -4.15)	(-1.89 - -0.55)	
																	
Depressive symptoms				0.16***				0.20***				0.09***				1.53***	
				(0.15 - 0.17)				(0.16 - 0.23)				(0.08 - 0.09)				(1.43 - 1.62)	
																	
Constant	4.46***	4.93***	7.43***	4.43***	15.44***	18.87***	24.32***	20.65***	2.79***	2.89***	3.97***	2.33***	56.05***	64.76***	82.86***	54.17***	
	(4.11 - 4.81)	(4.55 - 5.32)	(6.97 - 7.90)	(3.94 - 4.93)	(14.41 - 16.47)	(17.77 - 19.98)	(22.93 - 25.70)	(19.12 - 22.18)	(2.65 - 2.93)	(2.73 - 3.05)	(3.78 - 4.15)	(2.15 - 2.50)	(53.12 - 58.98)	(61.57 - 67.96)	(78.91 - 86.80)	(50.07 - 58.27)	
																	
Observations	5,000	5,000	5,000	5,000	5,000	5,000	5,000	5,000	5,000	5,000	5,000	5,000	5,000	5,000	5,000	5,000	
R²	0.07	0.10	0.17	0.29	0.13	0.19	0.22	0.24	0.13	0.15	0.24	0.45	0.10	0.15	0.20	0.33	
Unstandardized beta-coefficients; 95% confidence intervals in parentheses; *** p<0.001, ** p<0.01, * p<0.05, + p<0.10; sociodemographic covariates: gender, age, marital status, federal state, education, employment situation, migration background and religious affiliation; lifestyle-related covariates: frequency of sports activities, smoking behavior, and alcohol consumption; health-related covariates: self-rated health, and count of chronic conditions; mental health-related covariates: depressive symptoms; Federal state (with 16 states) is not shown here due to reasons of readability.  
