# Supplementary material for: Association of mainly vegetarian and vegan diets with loneliness, social isolation and social withdrawal in a German population survey
Source: PLoS One. 2026 Jul 15;21(7):e0353869. doi: 10.1371/journal.pone.0353869 (PMC13372175; doi:10.1371/journal.pone.0353869)
Supplement: S2 Table — Results based on multiple linear regressions (with bootstrapped standard errors). (DOC) [file pone.0353869.s002.doc]

S2 Table. Association of type of diet with loneliness, social isolation and social withdrawal. Results based on multiple linear regressions (with bootstrapped standard errors)
Independent variables	Loneliness	Objective social isolation	Perceived social isolation	Social withdrawal	
																					
Type of diet:																					
																					
- No vegetarian or vegan diet	Reference category	Reference category	Reference category	Reference category	Reference category	Reference category	Reference category	Reference category	Reference category	Reference category	Reference category	Reference category	Reference category	Reference category	Reference category	Reference category	Reference category	Reference category	Reference category	Reference category	
																					
- Mainly vegetarian diet 	0.11	0.04	0.08	0.14+	-0.09	-1.11***	-0.45*	-0.25	-0.08	-0.36+	0.21***	0.11***	0.12***	0.15***	0.02	2.59***	1.87**	2.28**	2.77***	0.56	
	(-0.05 - 0.26)	(-0.12 - 0.20)	(-0.08 - 0.24)	(-0.01 - 0.30)	(-0.23 - 0.06)	(-1.57 - -0.65)	(-0.90 - -0.00)	(-0.69 - 0.18)	(-0.51 - 0.35)	(-0.79 - 0.07)	(0.14 - 0.27)	(0.04 - 0.17)	(0.06 - 0.18)	(0.09 - 0.21)	(-0.03 - 0.07)	(1.19 - 3.99)	(0.48 - 3.25)	(0.91 - 3.65)	(1.42 - 4.12)	(-0.66 - 1.77)	
- Mainly vegan diet	0.58***	0.47**	0.46**	0.48**	0.20	-2.00***	-0.48	-0.08	-0.02	-0.36	0.46***	0.26***	0.24***	0.25***	0.10*	6.03***	4.35**	4.75***	4.87***	2.24*	
	(0.30 - 0.85)	(0.18 - 0.76)	(0.17 - 0.76)	(0.19 - 0.77)	(-0.07 - 0.47)	(-2.91 - -1.09)	(-1.35 - 0.38)	(-0.93 - 0.76)	(-0.87 - 0.82)	(-1.20 - 0.48)	(0.34 - 0.58)	(0.14 - 0.38)	(0.12 - 0.37)	(0.13 - 0.37)	(0.01 - 0.19)	(3.37 - 8.68)	(1.71 - 7.00)	(2.12 - 7.39)	(2.22 - 7.52)	(0.03 - 4.45)	
																					
Sociodemographic covariates																					
																					
Lifestyle-related covariates																					
																					
Health-related covariates																					
																					
Mental health-related covariates																					
																					
Constant	3.10***	4.46***	4.93***	7.43***	4.43***	15.66***	15.44***	18.87***	24.32***	20.65***	1.88***	2.79***	2.89***	3.97***	2.33***	36.89***	56.05***	64.76***	82.86***	54.17***	
	(3.04 - 3.16)	(4.10 - 4.82)	(4.54 - 5.33)	(6.96 - 7.91)	(3.92 - 4.95)	(15.47 - 15.85)	(14.43 - 16.44)	(17.76 - 19.98)	(22.94 - 25.70)	(19.11 - 22.19)	(1.86 - 1.91)	(2.66 - 2.92)	(2.74 - 3.04)	(3.79 - 4.14)	(2.16 - 2.49)	(36.36 - 37.41)	(53.11 - 58.99)	(61.52 - 68.01)	(78.91 - 86.80)	(50.15 - 58.19)	
																					
Observations	5,000	5,000	5,000	5,000	5,000	5,000	5,000	5,000	5,000	5,000	5,000	5,000	5,000	5,000	5,000	5,000	5,000	5,000	5,000	5,000	
R² 	0.00	0.07	0.10	0.17	0.29	0.01	0.13	0.19	0.22	0.24	0.02	0.13	0.15	0.24	0.45	0.01	0.10	0.15	0.20	0.33	
Unstandardized beta-coefficients; 95% confidence intervals in parentheses; *** p<0.001, ** p<0.01, * p<0.05, + p<0.10; sociodemographic covariates: gender, age, marital status, federal state, education, employment situation, migration background and religious affiliation; lifestyle-related covariates: frequency of sports activities, smoking behavior, and alcohol consumption; health-related covariates: self-rated health, and count of chronic conditions; mental health-related covariates: depressive symptoms
